# Anthrax vaccine design: strategies to achieve comprehensive protection against spore, bacillus, and toxin

**DOI:** 10.1186/1476-9433-4-4

**Published:** 2005-03-24

**Authors:** Julia Y Wang, Michael H Roehrl

**Affiliations:** 1 Channing Laboratory, Department of Medicine, Brigham and Women's Hospital, Harvard Medical School, Boston, MA 02115, USA; 2 Department of Biological Chemistry and Molecular Pharmacology, Harvard Medical School, Boston, MA 02115, USA

## Abstract

The successful use of *Bacillus anthracis *as a lethal biological weapon has prompted renewed research interest in the development of more effective vaccines against anthrax. The disease consists of three critical components: spore, bacillus, and toxin, elimination of any of which confers at least partial protection against anthrax. Current remedies rely on postexposure antibiotics to eliminate bacilli and pre- and postexposure vaccination to target primarily toxins. Vaccines effective against toxin have been licensed for human use, but need improvement. Vaccines against bacilli have recently been developed by us and others. Whether effective vaccines will be developed against spores is still an open question. An ideal vaccine would confer simultaneous protection against spores, bacilli, and toxins. One step towards this goal is our dually active vaccine, designed to destroy both bacilli and toxin. Existing and potential strategies towards potent and effective anthrax vaccines are discussed in this review.

## Anthrax as a biological weapon

*Bacillus anthracis*, the etiologic agent of anthrax, is an appealing biological weapon due to its high lethality and the ease of production and dissemination [1,2]. *B. anthracis *is a Gram-positive, aerobic, facultatively anaerobic, rod-shaped bacterium 1–1.5 μm wide and 3–10 μm long. An important aspect of *B. anthracis *is its ability to form dormant spores. Anthrax spores are naturally present in soil throughout the world, can remain viable for decades, and are highly resistant to adverse conditions such as heat, drought, ultraviolet light, gamma irradiation, and many disinfectants [3]. Anthrax spores are about 1–2 μm in diameter, optimal for inhalation and deposition in the alveolar spaces [4]. Inhalation of spores is almost always fatal, even with aggressive antimicrobial therapy [5]. Robust enough to withstand bomb detonation and small enough to aerosolize, anthrax spores may be easily dispersed over large populations by missiles, bombs, and aerosolization from flying aircraft. An aerosol release of odorless, invisible anthrax spores could travel far downwind from the point of release and cause catastrophic loss of life.

Natural cases of human inhalational anthrax infection are rare in recent history. In the century preceding the anthrax attack in 2001, only 18 cases of inhalational anthrax were reported in the U.S., with the most recent one in 1976. However, several lethal anthrax attacks have been documented. In 1979, anthrax spores were accidentally released from a military laboratory in the former Soviet city of Sverdlovsk. Sixty-four people were reported dead, although U.S. intelligence sources claimed the toll might have reached 1,000 [1]. In 2001, through deliberate delivery of anthrax spores in mailed letters, 10 confirmed cases of inhalational anthrax and 12 confirmed or suspected cases of cutaneous anthrax in humans were reported in the U.S. [6]. If handled less competently, this attack could have killed many more people. For example, the letter sent to Senator Daschle reportedly contained 2 g of *B. anthracis *powder, equivalent to 200 billion to 2 trillion spores [2]. The human LD_50 _(dose sufficient to kill 50% of infected persons) is estimated to be 2,500 to 55,000 inhaled spores, and as few as 1 to 3 spores may be sufficient to cause infection. Hence, an envelope full of anthrax spores, properly distributed, could kill many people.

## Disease and treatment

Anthrax can affect a wide variety of wild and domestic animals as well as humans [7]. Humans may become infected by anthrax spores through skin abrasions, ingestion, or inhalation [6]. Cutaneous anthrax is mild and can be treated with antibiotics. Gastrointestinal or inhalational anthrax, if left untreated, usually leads to fatal systemic disease with a mortality rate approaching 100 percent [6]. Inhalational anthrax is the most lethal form.

When anthrax spores are inhaled, they are deposited in alveolar spaces and ingested as inert particles by local macrophages [4,5]. The spores are then transported by the infected macrophages to mediastinal and peribronchial lymph nodes, where they germinate into vegetative bacilli. The bacilli escape from the macrophages and begin unimpeded extracellular multiplication within the lymphatic system, causing regional hemorrhagic lymphadenitis. The bacilli then spread into the bloodstream and continue rapid replication, reaching as many as 10^9 ^organisms per milliliter of blood [8]. All the while, the bacilli secrete high levels of exotoxins that intoxicate the host. The initial symptoms, such as malaise, fatigue, and cough, are nondescript and resemble those of influenza and other common upper respiratory infections, which makes early specific diagnosis difficult. After 2 to 5 days, there is a sudden onset of acute symptoms, which may include fever, chills, subcutaneous edema of the cheek and neck, widening of the mediastinum and pleural effusions, and hemorrhagic meningitis [4,5]. Death usually occurs within 24 hrs due to respiratory failure, with overwhelming bacteremia often associated with meningitis and subarachnoid hemorrhage [9].

Theoretically, there are several ways to approach anthrax: (i) vaccination to prevent disease development, (ii) elimination of spores, (iii) antibiotics to kill vegetative bacilli before the disease reaches a systemic stage, and (iv) conjunctive antitoxin therapy against anthrax toxin. Currently, treatment for anthrax relies mainly on antibiotics. A combination of antibiotics and aggressive hospital supportive care may succeed in the prodromal stage, but because the bacteria produce massive amounts of toxins that rapidly flood the blood and lymph system and send the patient into shock, the disease is often beyond treatment and inevitably fatal once the symptomatic stage has been reached [4,10]. Antitoxin therapy is also attractive, but if the bacteria continue to grow, will not be sufficient to stop anthrax. Full recovery from anthrax requires timely administration of antibiotics and antitoxic remedies, but timing is difficult due to non-specific initial symptoms. Hence, a prophylactic vaccine that prevents infection or stops infection at an early stage would be highly desirable.

## Vaccine against spores?

Critical steps in *B. anthracis *infection include spore entry and germination, bacillar multiplication and dissemination, and toxin production. Destroying the spores either before or after they enter host cells is an attractive strategy to prevent disease development. However, such an approach aimed either at control or treatment has thus far not been developed. This is partially due to our almost complete lack of molecular understanding of anthrax spores and the inability of our immune system to disable anthrax spores. Our knowledge about spores is essentially limited to descriptive electron-microscopic morphology dating back mostly to the 1960s. The anthrax spore consists of several morphologically distinct layers, from outside to inside: exosporium, spore coat, cortex, spore membrane, and core [11]. These structures jointly provide a highly protective "lockbox", protecting the core which houses the spore's genetic material [12]. Because of this robust architecture, anthrax spores are long-lived and extremely resistant to adverse environments. Once inside the host, the same structures allow anthrax spores to survive the host immune defense and to germinate.

It is not clear which type of immune response will be effective against spores. On the humoral immunity side, one could develop vaccines that elicit specific antibodies to opsonize spores. Several spore surface proteins have been identified recently [13-16]. However, since ingested, un-opsonized spores germinate in and are not killed by macrophages, would opsonized spores be taken up by macrophages or other phagocytes and be killed [17]? Would complement be able to lyse the spores? On the cellular immunity side, could cytotoxic T cells destroy the dormant or germinating spores inside macrophages? Consequently, whether a conventional vaccine design with the goal of eliciting antibodies or T cell immunity would be effective against spores is still an open question. By contrast, development of vaccines targeting the anthrax exotoxin and extracellular bacilli has been much more promising.

## Two major virulence factors

The virulence of *B. anthracis *is attributable to two major factors: a poly-γ-D-glutamic acid (PGA) capsule and a secreted tripartite protein complex toxin. Fully virulent strains of *B. anthracis *carry two large extrachromosomal plasmids, pXO1 and pXO2, which encode for the toxin and the PGA capsule [18,19], respectively. Absence of either plasmid results in a marked reduction in virulence [7,20].

The first description of the *B. anthracis *capsule dates back to 1903 and M'Fadyean's discovery of the relationship between in vivo formation of the capsule and virulence [21]. The importance of the capsule was further emphasized in the 1950s when Bail demonstrated that strains that had lost the ability to produce a capsule were avirulent [7]. Wild-type anthrax bacillus is capsulated in animal hosts, but not in culture unless suitable conditions are provided [7]. Factors that influence capsule formation are important for determining the outcome of infection. In susceptible animals, the bacilli remain encapsulated, whereas in resistant animals, the capsule is shed [22]. PGA is an anionic, poorly immunogenic polypeptide that disguises the bacteria from the host immune surveillance and, by virtue of its negative charges, inhibits bactericidal activity by the host [7]. Thus, the PGA capsule allows virulent anthrax bacilli to grow virtually unimpeded in the infected host.

Anthrax toxin, the other major virulence factor of *B. anthracis*, was discovered in 1954, when Smith and Keppie demonstrated that sterile plasma from experimentally infected guinea pigs was lethal when injected into other animals [8,23]. Anthrax toxin includes lethal toxin and edema toxin, which are binary complexes formed, respectively, by lethal factor (LF) and edema factor (EF) with protective antigen (PA) [24,25]. These proteins are released discretely as nontoxic monomers. The characteristic edema observed in cutaneous anthrax is produced by edema toxin [10,22]. EF is a calmodulin-dependent adenylate cyclase and catalyzes the production of intracellular cyclic AMP from host ATP [26]. Increased cellular levels of cyclic AMP upset water homeostasis and can cause massive edema. The most severe symptoms of anthrax, such as hypotension, shock, and death, are caused by lethal toxin [10,22]. LF is a zinc metalloprotease that inactivates mitogen-activated protein kinase kinase and acts specifically on macrophages [27-29]. Lethal toxin, when given intravenously, is relatively weak compared with other bacterial toxins [9]. Both lethal and edema toxins are thought to be important in the establishment of disease by impairing the host defenses.

Overall, the capsule and toxin act jointly to maximize the survival of bacilli in the host. While the PGA capsule passively protects the bacilli from host defense, the toxin actively impairs the host to further ensure a favorable environment for bacillar growth.

## Veterinary vaccine: live attenuated organisms

Studies on the vaccination of animals against anthrax date back to the end of the 19th century. In the 1870s, Robert Koch established *B. anthracis *as the etiologic agent of anthrax. In 1881, Pasteur demonstrated protective immunization against anthrax using a heat-attenuated strain, which was later recognized as an encapsulated strain with reduced virulence [7,21,30]. In the 1930s, Sterne developed an attenuated, toxigenic, but non-encapsulated strain that proved to be remarkably effective as a vaccine for domestic animals and is now used worldwide [7]. Although effective, the attenuated spore vaccines developed by Pasteur and Sterne suffer from declining potency and troublesome variations in virulence that led occasionally to the death of animals [21,30]. Due to residual virulence, the attenuated spore vaccines are not used in humans.

## Existing human vaccine: crude preparation of PA

The development of anthrax vaccines for human use began in the 1940s, motivated by fear of the use of anthrax as a biological weapon. In 1970, the protective antigen (PA)-based cell-free subunit vaccine, designated "anthrax vaccine adsorbed" (AVA) or Biothrax, was licensed and recommended for use by a small population of mill workers, veterinarians, laboratory scientists, and others with risk of occupational exposure to anthrax [30,31]. Increased concern about the use of anthrax in warfare led the Department of Defense to vaccinate U.S. military personnel in the 1990s. 

AVA is prepared from microaerophilic cultures of the attenuated, non-encapsulated strain V770-NP1-R of *B. anthracis *[9,31]. Downstream processing begins with filtration, which removes the bacterial cells along with some EF and LF. The cell-free culture filtrate, thought to contain predominantly PA, is then adsorbed to aluminum hydroxide [32]. Small amounts of formaldehyde and benzethonium chloride are added as preservatives [9]. Although PA is by itself an effective immunogen, it is not clear whether the small amounts of LF and EF that may be present in some lots contribute to the vaccine's effectiveness. A vaccine licensed in the UK is prepared by alum precipitation of the sterile culture filtrate of a derivative of the attenuated Sterne strain. It contains higher amounts of LF and EF than AVA [33,34].

The efficacy of AVA and highly purified PA preparations have been tested in various animal models, including mice, hamsters, guinea pigs, rabbits, and monkeys [31,32,35]. Interestingly, protection varies widely among species. AVA does not protect hamsters at all [36]. In mice, the PGA capsule appears to be the primary virulence factor, and PA-based vaccines confer only limited protection [37]. There is no direct correlation between anti-PA titers and protection in mice and hamsters [36,37]. In guinea pigs, AVA provides partial protection [35,38,39], but AVA appears to be more effective in rabbit and macaque models [35,40-42]. It is noteworthy that "efficacy", as defined in these studies, is relative. When macaques were exposed experimentally to doses of up to 900 times the LD_50_, 88–100% of the animals were protected [31]. However, simulation studies suggest that a person opening a letter filled with anthrax spores and standing over it for 10 min could inhale up to 3,000 times and perhaps as much as 9,000 times the LD_50 _for humans [2].

A controlled human trial was conducted in the 1950s with a vaccine similar to AVA but derived from a different attenuated, non-encapsulated strain of *B. anthracis *grown aerobically. In a susceptible population of textile mill workers in the northeastern states of the US who processed occasionally contaminated goat hair, vaccination provided 92.5% protection against cutaneous anthrax [43,44]. However, no assessment of inhalational anthrax could be made, because cases were too few.

There are several concerns regarding AVA: (i) It does not protect all animal hosts against different strains of *B. anthracis*. AVA or PA-based vaccines in general induce toxin-neutralizing antibodies. The mechanism underlying the protective action of PA-based vaccines is unclear. It is thought that anti-PA vaccines protect the host from intoxication and thus allow the immune system to deal with the organism. However, evidence that primates vaccinated with AVA or PA vaccine develop transient episodes of bacteremia suggests that vaccination does not prevent the growth of bacilli. (ii) The administration of AVA is burdensome, requiring subcutaneous injections at 0, 2, and 4 weeks and 6, 12, and 18 months with subsequent yearly boosters [31,32]. In a field trial of a vaccine similar to AVA, one case of cutaneous anthrax occurred 5 months after the initial 3-dose series and just before the scheduled 6-month booster [9]. This case suggests that immunity is not long-lasting and that frequent boosters may be necessary. (iii) The preparative processing of AVA is crude and lacks consistency. Furthermore, there are relatively high rates of local and systemic adverse reactions, likely due to residual toxicity in AVA or other contaminants.

## Improvement: highly purified recombinant PA

The limitations of AVA have raised widespread interest in developing improved anthrax vaccines consisting of well-characterized components. A new generation of vaccines based on highly purified recombinant PA is currently being developed and evaluated. There have been numerous attempts to establish high-level PA expression systems based on a variety of organisms, including attenuated strains of *B. anthracis*, *B. subtilis*, *B. brevis*, *Salmonella typhimurium*, *E. coli*, viruses, insect cells, and plants [45-50]. In addition, genetic immunization with DNA encoding for PA is being explored [51].

The highly purified PA vaccines are expected to induce essentially the same immunity as AVA. While some disadvantages of AVA due to its "dirty" preparation may be overcome, limitations in protection and lack of an immune memory response may be intrinsic to PA itself.

New strategies are needed for further improvement. One possibility is that other antigens or cellular immunity in addition to PA-specific antibodies are required for full protection in different animal species. This is supported by studies showing that the live veterinary vaccine provides significantly greater protection against anthrax in experimental animals than does AVA, despite the fact that it frequently induces lower levels of antibodies to PA [33,39,42,52,53]. After a naturally acquired infection, and depending on when samples are taken, 68–93% of cases develop antibodies to PA, 42–55% of cases develop antibodies to LF, and antibodies to EF are less frequently detected [34,54-56]. Interestingly, antibodies to the capsule are detected in 67–94% cases [55,56], whereas no response to the capsule is expected in the vaccinees who have been vaccinated with AVA or non-encapsulated live vaccines.

## Further improvement: two-in-one postexposure antitoxic therapy/vaccine

Post-exposure vaccination is the most likely scenario, given the rarity of natural inhalational anthrax infection. However, the use of PA as a postexposure vaccine may be limited. Since PA is a natural component of anthrax toxin and may contribute to toxin formation, it may not be safe to administer a PA-based vaccine to persons who have been or are suspected of having been exposed to anthrax. We recently proposed the replacement of PA in vaccines with a dominant-negative inhibitor (DNI) of anthrax toxin [57]. DNI is a translocation-deficient mutant of PA carrying double mutations of K397D and D425K and has been demonstrated to interfere with the intoxication process, providing immediate therapeutic protection against anthrax toxin in vivo [58,59]. Furthermore, when used as a vaccine, DNI is more immunogenic than PA [57].

The symptoms and incubation period of human anthrax vary depending on the route of transmission. The reported incubation period of inhalational anthrax, the most lethal form, ranges from 1 to 43 days [60]. Data from animal studies suggest that anthrax spores persist in the host for several weeks after infection and that antibiotics can prolong the incubation period for developing disease. Studies in nonhuman primates indicate that inhaled spores do not immediately germinate within the alveolar recesses but reside there, possibly for weeks, until taken up by alveolar macrophages [61]. Development of anthrax disease can be prevented as long as a therapeutic level of antibiotic is maintained to kill germinating bacilli. After antibiotics are discontinued, disease will develop if the remaining spores germinate in the absence of a protective immune response. In previous animal studies, treatment with antibiotics for 5 or 10 days, beginning one day after anthrax spore aerosol challenge, was protective during drug therapy, but animals died after the antibiotic was discontinued [61]. Longer antibiotic treatment, e.g., for 30 days, might be necessary to ensure full recovery. However, antibiotic treatment cannot protect against relapse or subsequent exposure to anthrax. Long-term protection was achieved only by combining antibiotic therapy with post-exposure vaccination [62]. In such situations, conjunctive antibiotic treatment and vaccination with DNI would be ideal, whereas administration of PA would be potentially dangerous because it may combine with trace amounts of LF or EF and cause toxicity.

During the anthrax attack in the U.S. in 2001, some groups of individuals underwent a 60-day regimen of antibiotic prophylaxis. Statistical analysis shows that this preventative measure may have saved many lives [63]. In the event of an anthrax attack, the incubation period will vary among individuals. The timely post-exposure administration of DNI as both a conjunctive therapy to antibiotics and a prophylactic vaccine would be expected to be superior, both for individual and public health perspectives.

## Enhanced antigen processing through "endosomal trapping"?

**Figure 1:**
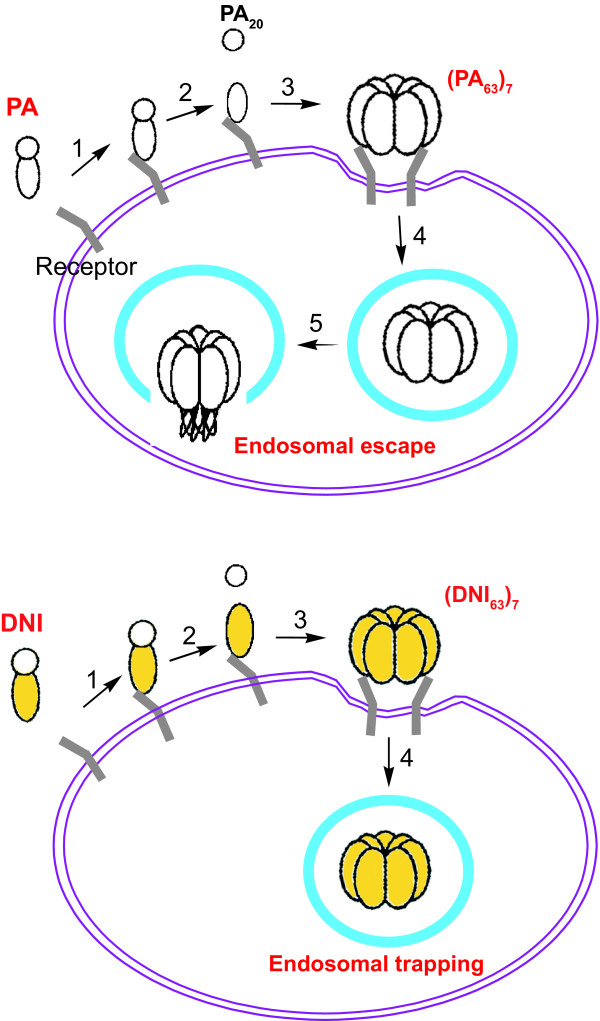
Models illustrating the different cellular fates of PA and DNI. Top: Upon binding cellular receptors (step 1), PA is cleaved (step 2). The small fragments diffuse away, and the cell-bound fraction self-assembles into heptameric cores termed (PA_63_)_7 _(step 3). The heptamers then undergo receptor-mediated endocytosis (step 4). Once inside the acidic endosomal compartment, the PA heptamers change conformation and insert into the endosomal membrane (step 5). Bottom: DNI, similar to PA, enters the endosome (steps 1–4). However, DNI heptamers do not undergo the necessary conformational changes for insertion into the membrane and therefore remain trapped inside the endosome.

The increased immunogenicity of DNI not only recommends it as a potential new anthrax vaccine but may also point to a potentially important general strategy for future vaccine design. We proposed an "endosomal trapping" mechanism that rationalizes more efficient antigen processing of DNI over native PA [57]. As illustrated in Figure 1, PA molecules undergo several transformations during the intoxication process [24]. Upon release, the 83-kD PA molecule first binds to a receptor present on most mammalian cells [25,64-66]. The cell-bound PA is then cleaved by furin or a furin-like protease into two components [67]. The dissociation of the N-terminal 20-kDa fragment PA_20_ exposes a binding site for LF or EF to the cell-bound 63-kDa fragment PA_63 _and also enables PA_63 _to assemble into a heptameric, ring-shaped prepore (PA_63_)_7 _[68]. LF or EF bind competitively to (PA_63_)_7 _with very high affinity (*K*_d _~ 1 nM) [69,70]. The complex is internalized by receptor-mediated endocytosis and trafficked into an acidic endosomal compartment. (PA_63_)_7 _undergoes a major conformational rearrangement following the pH change and forms a membrane-spanning β barrel that enables its penetration into the cytosol [71,72]. By means of (PA_63_)_7_, EF and LF translocate into the cytosol, where they modify substrates and exert toxic effect (see above). However, the two mutations in DNI inhibit the required conformational change of (DNI_63_)_7 _or chimeric DNI/PA heptamers from a ring-shaped core to a β barrel and thus prevent the heptamer from inserting into the endosomal membrane [59]. Consequently, these mutations inhibit the translocation of LF or EF into the cytosol and prevent cytotoxicity [58].

Protein antigens are commonly processed in the endosomes of antigen-presenting cells and elicit T-cell help in antibody production. The peptides from degraded protein antigens are loaded onto MHC class II molecules and transported to the cell surface. Thus, the insertion of PA pores in the endosomal membrane may disrupt the integrity of the endosome and thus compromise its function, perhaps leading to altered vesicular trafficking of endocytic PA molecules. Altered vesicular trafficking of PA may affect its delivery to specialized endosomal compartments, where antigenic processing of PA occurs. It is also possible that PA pore formation might simply change the pH or ionic environment inside the lumen of the endosome and thus affect the processing/presentation machinery. Furthermore, PA may disrupt or otherwise "leak" out of the endosome and simply enter the cytosol (as is the case for the LF and EF subunits) and thus escape efficient endosomal processing. In contrast, DNI heptamers do not insert into the endosomal membrane and block translocation of LF and EF into the cytosol. Thus, DNI is expected to be trapped in the endosome and undergo normal vesicular trafficking. Hence, the translocation-deficient mutant DNI may concentrate in the endosome; that is, DNI may be more prone to "endosomal trapping" than PA, and this difference in localization may increase the generation of processed DNI peptides suitable for binding to MHC class II molecules. If such a mechanism indeed explains the enhanced immunogenicity of DNI, similar strategic mutations might be introduced into other toxin immunogens to enhance their immunogenicity via this "endosomal trapping" mechanism.

## New-generation vaccines: dual protection against bacilli and toxin

Despite the direct toxicity of anthrax toxin, systemic anthrax disease is amplified by the massive extracellular replication of the bacilli that produce it. Directly targeting *B. anthracis *at the extracellular stage is both appealing and feasible. It should be reiterated that it is the bacilli that are replicating and secreting toxins, and eliminating bacilli would abrogate toxin production at the source. A major virulence factor of *B. anthracis *is its anti-phagocytic PGA capsule [7,73]. The role of a capsule in virulence has been well established for numerous bacterial species, such as *Streptococcus pneumoniae *and type b *Haemophilus influenzae *(Hib) [74,75]. In these bacteria, the capsule also confers resistance to phagocytosis by host cells. The immunological role of the *B. anthracis *PGA capsule very much resembles the role of capsular polysaccharides in other bacteria. The weakly immunogenic capsules do not favor an immune response but rather enable encapsulated bacteria to evade the host immune defenses. Vaccines based on capsular polysaccharides have been highly successful against pathogens such as Hib. We hypothesized that PGA-based vaccines could have similar success in protection against *B. anthracis *infection.

Although capsular PGA is a promising antigen for a new anthrax vaccine, PGA alone has limited use because of its weak immunogenicity. Fortunately, similar to polysaccharides, the immunogenicity of PGA can be significantly enhanced by conjugation to a strongly immunogenic protein carrier [76,77]. Functional studies have demonstrated that anti-PGA antibodies do indeed confer protection by mediating opsonophagocytosis of *B. anthracis *[78,79].

Because the pathogenesis of anthrax is largely attributable to replication of bacilli and release of toxin, we constructed a new generation of anthrax vaccines by chemically conjugating PGA and PA, the two virulence components of *B. anthracis*. This dually active anthrax vaccine (DAAV) is capable of inducing high levels of specific antibodies to both capsule and toxin [76]. We envision that a PGA-directed antibody response will achieve protection against anthrax by eliminating bacteria early in the sequence of infection, well before the onset of bacteremia and toxemia. The fact that the very early stages of anthrax disease can be treated by antibiotics also supports the notion that an anti-bacillar vaccine may be effective, or at least a valuable addition to vaccines based on PA alone. In addition, antibodies to PA provide a parallel line of defense against residual toxin. DAAVs embody the paradigm of combining both antibacterial (i.e., prophylactic) and antitoxic (i.e., therapeutic) components into a single vaccine.

## Authors' contributions

Julia Y. Wang and Michael H. Roehrl jointly wrote the paper.
